# Radiological Manifestation of Neurological Complications in the Course of SARS-CoV-2 Infection

**DOI:** 10.3389/fneur.2021.711026

**Published:** 2021-10-20

**Authors:** Katarzyna Sklinda, Małgorzata Dorobek, Piotr G. Wasilewski, Karol Dreżewski, Marta Dȩbicka, Jerzy Walecki, Bartosz Mruk

**Affiliations:** ^1^Centre of Postgraduate Medical Education, Department of Radiology, Central Clinical Hospital of the Ministry of Interior, Warsaw, Poland; ^2^Department of Neurology, Central Clinical Hospital of the Ministry of Interior, Warsaw, Poland

**Keywords:** COVID-19, SARS-CoV-2, neurological complications, radiological image, CT, MRI

## Abstract

Many reports suggest the SARS-CoV-2 infection may result in neurological complications. A wide spectrum of clinical syndromes have been reported, including both central and peripheral nervous system. Such symptoms may be a consequence of a direct viral injury, secondary to systemic inflammatory response, autoimmune processes, ischemic lesions or combination of these. Anosmia and dysgeusia are highly prevalent in the early stage of infection. Cerebrovascular events in patients with COVID-19 have also been documented with increasing frequency. Some cases of parainfectious autoimmune neurologic manifestations concurrent with active SARS-CoV-2 infection have been described, including hemorrhagic necrotizing encephalopathy, Guillain-Barré and Miller-Fisher syndromes. There are also a few reports documenting encephalitis and acute demyelinating encephalomyelitis (ADEM) in the course of COVID-19. There is also a growing number of cases of patients after recovery from COVID-19 with psychosomatic disorders, manifesting with memory disfunction, cognitive functions disorders, depression or other affective disorders, which may lead to a decrease of intellectual functions. Many of these neurological manifestations of the infection are possible to distinguish using radiological imaging techniques. It plays a very important role in evaluating the course of COVID-19 as well as diagnosing respiratory complications and choosing a proper management of infected patients. Similarly, radiological techniques play crucial role in identifying the cause of neurological symptoms connected to SARS-CoV-2 infection, being one of the most important elements of diagnostics. Especially in case of the presence of nervous system implication, using radiological imaging techniques to monitor the emerging onset of various symptoms is crucial to assess the severity and scope of involvement. Quick diagnostic process and identifying complications as fast as possible in order to implement specific treatment can be crucial to avoid long-term secondary conditions and accelerate the recovery period. In this review, we present the most important neurological complications that may occur in the course of SARS-CoV-2 infection and summarize their radiological manifestations.

## Introduction

First reported in Wuhan, China, COVID-19 predominantly presents with respiratory symptoms and fever. However, it is known to have several atypical manifestations.

Many reports suggest that the infection may result in neurological complications. A wide spectrum of clinical syndromes has been reported, including both central and peripheral nervous system.

Such symptoms may be a consequence of a direct viral injury, secondary to systemic inflammatory response, autoimmune processes, ischemic lesions, or combination of these (1, 2).

The largest study to date analyzing 214 patients of Wuhan hospitals shows that neurological symptoms were present in 36.4% of cases, of which dizziness and headache were most prevalent (3).

Anosmia and dysgeusia are common in early stages of the disease and have been considered evidence of hypothetical olfactory tract invasion (4).

Another concerning fact is a correlation of COVID-19 to cerebrovascular events, such as stroke, that affects ~1–3% of hospitalized patients (5).

Moreover, autoimmune-based manifestations such as acute disseminated encephalomyelitis, hemorrhagic necrotizing encephalopathy (6), Guillain–Barré syndrome, and Miller–Fisher syndrome (MFS) have also been observed in patients with SARS-CoV-2 infection (2, 7).

Radiological imaging plays a crucial role in evaluating the course of COVID-19 as well as diagnosing respiratory complications and choosing a proper management of infected patients.

Similarly, radiological techniques play a crucial role in identifying the cause of neurological symptoms connected to SARS-CoV-2 infection, being one of the most important elements of diagnostics.

Magnetic resonance imaging (MRI) and computed tomography (CT) are the best radiological methods to visualize the effects of the infection on the nervous system.

In this review, we present the most important neurological complications that may occur in the course of SARS-CoV-2 infection and their radiological manifestations.

## Neurological Involvement in the Course of Covid-19

It is well-documented that COVID-19 is as much of a respiratory disease as a neurological one.

The mechanism of infection has been thoroughly explained and proved.

SARS-CoV-2 virions are equipped with a viral structural spike (S) protein that binds to the angiotensin-converting enzyme 2 (ACE2) receptor on human cells.

There is high expression of the ACE2 receptor in multiple organs, including lung epithelial cells, but also in heart, kidney, pancreas, spleen, gastrointestinal system, bladder, cornea, and blood vessels (8, 9).

The ACE2 receptor is also found in the central and peripheral nervous systems and in skeletal muscle (10, 11).

Viral replication within human host cells is followed by viral release through cell destruction (8, 9). Moreover, SARS-CoV-2 activates an inflammatory response that can result in a cytokine storm and ultimately multi-organ injury (8, 9).

However, the origins of neural involvement is not yet fully uncovered. There are a couple of presumable mechanisms of neural involvement during SARS-CoV-2 infection.

First of all, a known neurotropism of previous SARS-CoV strains is the basis of the direct central nervous system (CNS) spread theory. The virus could access the CNS through olfactory pathways or the bloodstream, causing meningitis and encephalitis (12, 13).

The involvement of the respiratory center in the brainstem can explain the well-documented rapid respiratory aggravation with marked hypoxia despite a lack of symptomatic dyspnea (14).

Another hypothesis is the indirect involvement of neurological tissue due to an excessive systemic inflammatory response. In this case, an uncontrolled immune reaction is the cause of widespread dysregulation of homeostasis with coagulopathy. It may also increase the risk of acute cerebrovascular diseases (CVDs) (15, 16).

Several autoimmune-based neurologic complications such as acute disseminated encephalomyelitis and Guillain–Barré syndromes, as known parainfectious syndromes, can also be considered as a straight outcome from a microbial infection (6, 17, 18).

Complications may concern both central and peripheral nervous system and occur with different frequencies. According to data from China, presence of various neurological symptoms reached 36.4% of hospitalized patients (19). Other data showed that occurrence of such complications reached even more than 60% patients.

Symptoms from the nervous system may appear during the acute phase or the subacute phase or even show up in the later stages of the infection. Moreover, they may even precede the symptoms from the respiratory system.

Anosmia and dysgeusia are highly prevalent in the early stage of infection (20). It has been proposed as a support of the hypothesis of CNS spread *via* the olfactory tract (21).

Cerebrovascular events in patients with COVID-19 have also been documented with increasing frequency (22, 23).

Some cases of parainfectious autoimmune neurologic manifestations concurrent with active SARS-CoV-2 infection have been described, including hemorrhagic necrotizing encephalopathy (6), Guillain–Barré syndrome, and MFS (6, 17, 18, 24, 25).

There are also a few reports documenting encephalitis and acute demyelinating encephalomyelitis (ADEM) in the course of COVID-19.

There is also a growing number of cases of patients after recovery from COVID-19 with memory dysfunction, cognitive functions disorders, depression, or other affective disorders, which may lead to a decrease of intellectual functions.

Many neurological manifestations of the infection mentioned above are possible to distinguish using radiological imaging techniques. However, nearly all published neuroradiological findings in COVID-19 lack specificity.

Nevertheless, quick diagnostic process and identifying complications as fast as possible in order to implement specific treatment can be crucial to avoid long-term secondary conditions and accelerate the recovery period.

In the next section, we present the most notable neurological complications in the course of COVID-19 and their presentation that can be obtained using radiological imaging.

## Manifestation of Central Nervous System Complications in Radiological Findings

### Acute Cerebrovascular Disease

Acute cerebrovascular events are the most common complication of COVID-19 involving the central nervous system and were reported in 0.5–5.9% of infected patients (23, 26). The most common type of CVD was the acute ischemic stroke. The median time to onset of CVD was 9 days.

Patients with severe onset of the disease were more at risk of developing the acute CVD than the ones with a mild infection. In this group of patients, the reported range of the acute CVD was 0.8–9.8% (23).

The scope of ischemic stroke, hemorrhagic stroke, and cerebral venous thrombosis according to studies ranged from 0.4 to 4.9%, from 0.2 to 0.9%, and from 0.3 to 0.5%, respectively (23, 27).

**Figure 1:**
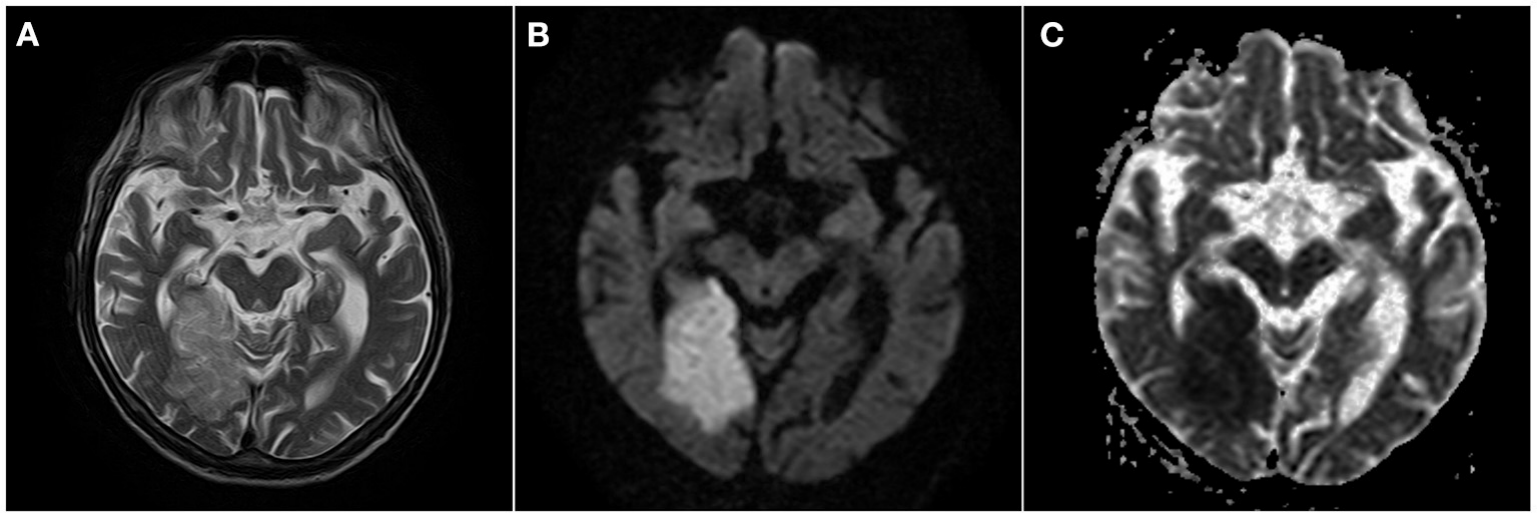
Acute ischemic stroke in the right occipital lobe. Hyperintense area of edema is seen on T2-weighted image **(A)**. Diffusion restriction seen as marked hyperintensity on DWI **(B)** and reduced ADC values **(C)**.

In Figure 1, we present an ischemic stroke in MR examination of a patient with COVID-19 from our department (Figure 1).

Various reports suggest that SARS-CoV-2 infection predisposes patients to an ischemic stroke and increases the risk especially of a cryptogenic stroke. Incidence of such event coupled with COVID-19 ranges between 2.5 and 6.4%.

Also, there are multiple case reports and studies documenting aneurysmal (28) and non-aneurysmal subarachnoidal hemorrhage (SAH) (29), deep cerebral venous thrombosis (CVT) (30), and CNS vasculitis (31) in COVID-19 patients.

**Figure 2:**
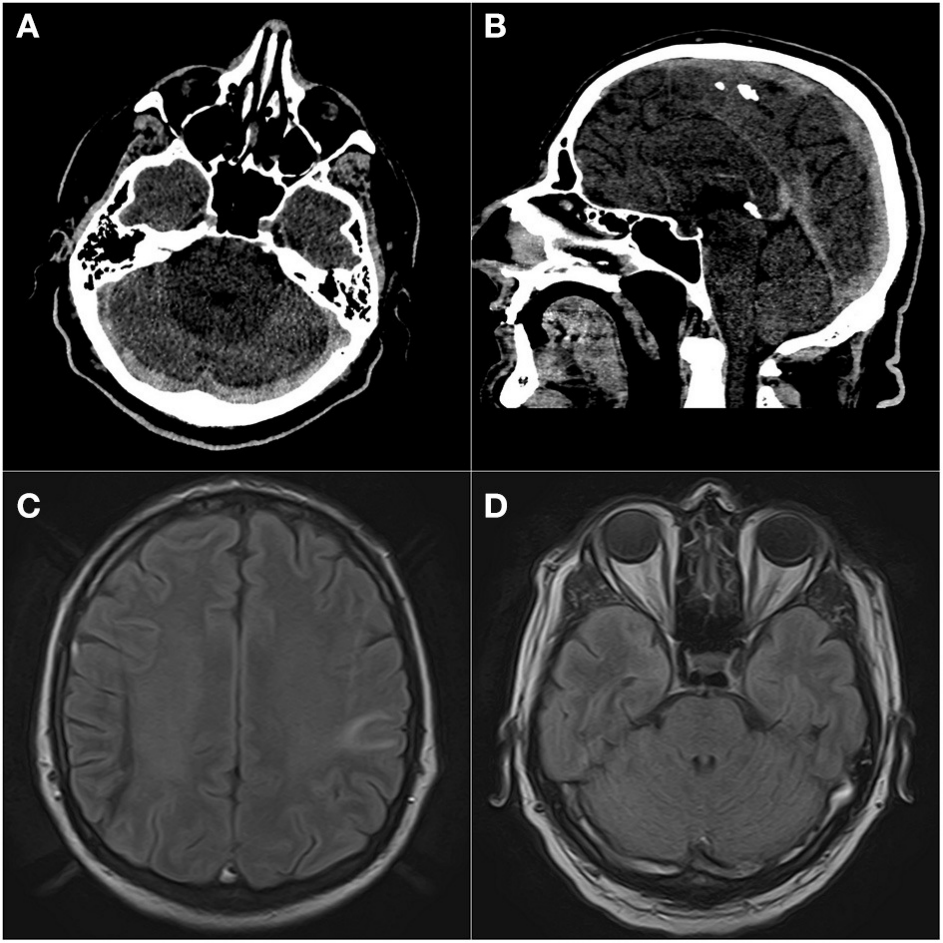
Massive dural sinus thrombosis. Prominent hyperattenuation of transverse **(A)**, superior sagittal, and straight sinuses **(B)** on non-contrast CT. FLAIR images **(C,D)** of the same patient show abnormal high signal in dural sinuses and hyperintense area in the left parietal lobe consistent with venous infarct. Patient presented with impaired consciousness.

In Figure 2, we present an example of CVT captured in MRI of a COVID-19 patient from our department (Figure 2).

Acute ischemic stroke is the most common neuro-radiologic abnormality seen among COVID-19 patients with neurological complications (60–65% of the cases) (4).

### Meningoencephalitis, Encephalopathy, and Disruptions of Consciousness

Encephalitis, meningitis, and encephalopathy were reported as complications in COVID-19 patients. However, only several cases of such complications have only been documented.

The frequency of encephalitis presented in two retrospective studies was 0.03% (32) and 0.1% (33).

A couple of retrospective studies reported seizures with the frequency ranging from 0.5 to 1.4% (19, 34). Cases of all types of seizures were reported in patients with COVID-19.

Meningitis can be observed in MRI examination. In T1 images, the presentation may be normal; however, sulci may appear less hypointense than normal. After applying gadolinium contrast, a pachymeningeal enhancement can be observed.

**Figure 3:**
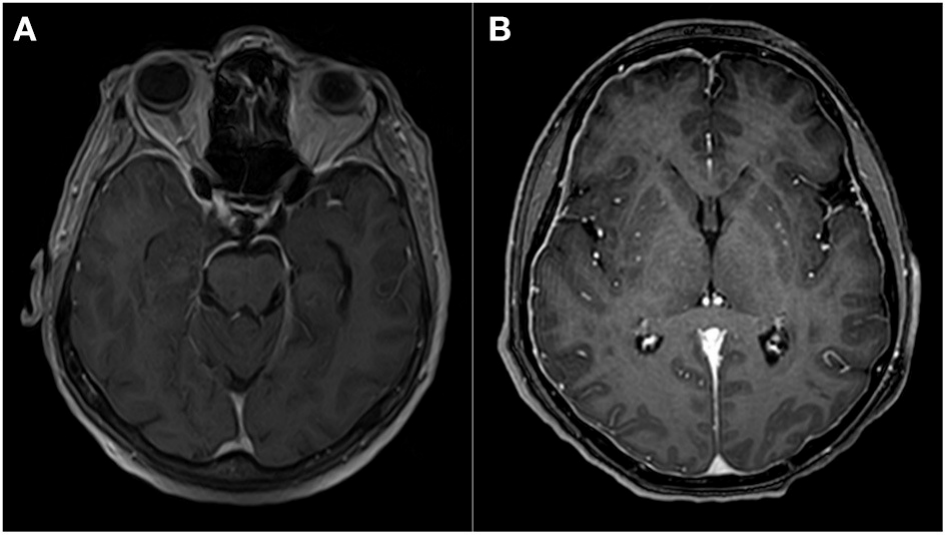
T1 contrast-enhanced images **(A,B)** show pachymeningeal enhancement in two different patients, indicative of meningitis. In case **(A)**, it was an isolated finding, while in case **(B)**, coexisting white matter lesions suggestive of acute demyelination were present.

In Figure 3, we present a manifestation of meningitis in MRI of a patient with an ongoing SARS-CoV-2 infection from our department (Figure 3).

### Posterior Reversible Encephalopathy Syndrome

Posterior reversible encephalopathy syndrome (PRES) as a complication of COVID-19 has been reported in studies (34).

It is a neurotoxic state that occurs due to the impaired autoregulation to response to acute changes in blood pressure of the posterior circulation. Hyperperfusion and, as a result, disruption of the blood–brain barrier emerge in vasogenic edema (commonly without infarction), most usually in the parieto-occipital regions.

Single cases of PRES in a postpartum period were also documented (35).

There are also case reports describing patients with transient cortical blindness in a PRES-like syndrome in the course of infection (36).

In patients with PRES, MRI examination shows bilateral T2/FLAIR hyperintensities in affected areas, usually in occipital and parietal lobes and diffusion restriction in DWI sequences due to edema. Such abnormalities may disappear in control examinations after a few weeks.

### Acute Disseminated Encephalomyelitis (ADEM)

It is a rare acute inflammation and demyelination of white matter that typically follows a recent viral infection or vaccination. Usually, gray matter is also involved, especially basal ganglia, but to a lesser extent than white matter. Similarly, the spinal cord can also be affected.

There are only a few documented cases of ADEM in the course of COVID-19 (37, 38).

In MRI, ADEM is observed In T2 images as regions of high signal, with surrounding edema typically situated in subcortical locations. The thalami and brainstem can also be involved.

After administering gadolinium contrast medium, a punctate, ring, or arc enhancement is often demonstrated along the leading edge of inflammation in T1 C+ images.

**Figure 4:**
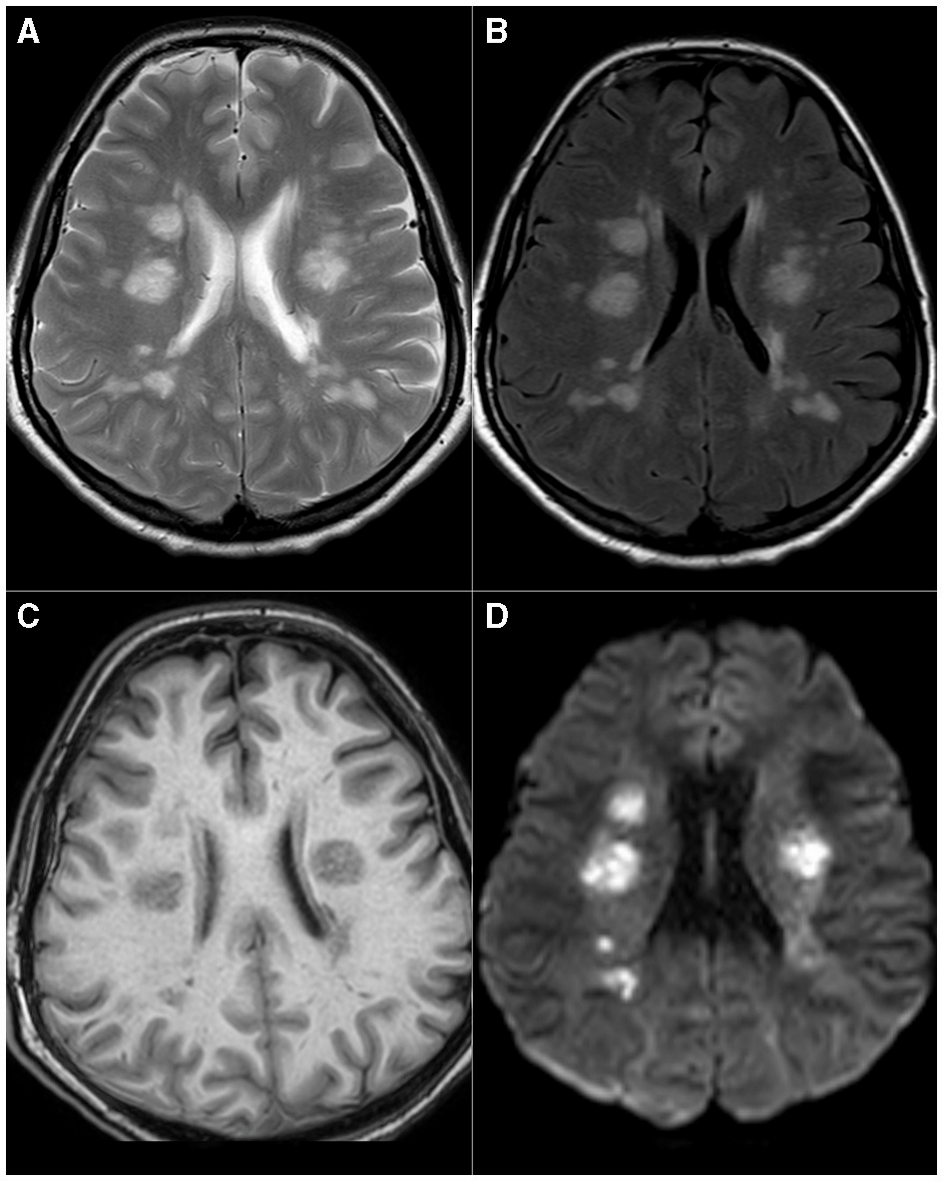
Acute disseminated encephalomyelitis (ADEM). Multiple, bilateral T2 **(A)** and FLAIR **(B)** hyperintense foci present in periventricular areas of white matter, hypointense, or invisible on T1-weighted images **(C)**. These lesions show pronounced diffusion restriction seen as high-signal intensity on DWI images **(D)**.

In Figure 4, we present a series of images from MRI from the single patient with ADEM in the course of COVID-19 who was treated in the Department of Neurology of our hospital (Figure 4).

### Multiple Sclerosis (MS)

SARS-CoV-2 infection may be related not only to ADEM development but also to unmask or trigger multiple sclerosis. The relationship between other coronaviruses and foci of demyelination within the central nervous system has been known (39, 40). Few reports describe the development of multiple sclerosis symptoms during the active phase of SARS-CoV-2 infection or during the patient's recovery from COVID-19-related symptoms (41, 42).

MRI may show contrast-enhancing as well as non-enhancing white matter lesions in juxtacortical, periventricular, and infratentorial locations and signs of optic neuritis (41, 42). SARS-CoV-2 PCR was not detected in the CSF in the cases mentioned above, suggesting against direct viral invasion causing those syndromes (41, 42).

Also, the impact of SARS-CoV-2 infection on the risk of the relapse of multiple sclerosis remains unknown. One retrospective study conducted in a relatively small group of patients has shown that COVID-19 increases the risk of multiple sclerosis relapse (43). This finding is consistent with other studies showing an increased risk of MS exacerbation associated with other upper respiratory tract infections, 10–30% of which are caused by coronaviruses (44–46).

### Hemorrhagic Necrotizing Encephalopathy

Acute hemorrhagic necrotizing encephalopathy is a rare type of encephalopathy characterized by multiple bilateral brain lesions, mainly involving the thalami, but also the putamina, internal and external capsules, cerebellar white matter, and the brainstem tegmentum.

It is usually triggered by several viral infections, most notably influenza A, influenza B, parainfluenza, varicella, and enterovirus.

Recent studies show that the SARS-CoV-2 is also a possible cause of such complication (6).

On CT scanning, the corresponding thalamic, putamina, cerebral, cerebellar, and brainstem abnormalities are hypodense. Intracranial hemorrhage and cavitation can be seen.

In MR images, a bilateral symmetrical thalamic involvement can be observed. Abnormal signals on MRI are hypointense on T1 and hyperintense on T2. Restricted diffusion in DWI sequences of the involved regions can be seen. Hemorrhage, cavitation, and post-contrast enhancement are also noted.

### Bickerstaff Brainstem Encephalitis

Bickerstaff brainstem encephalitis (BBE) is a rare immune-mediated condition characterized by a triad of clinical symptoms—ophthalmoplegia, ataxia, and consciousness disruption.

Bickerstaff encephalitis is usually observed after varicella zoster virus (VCV) and cytomegalovirus (CMV) infections. Most recently, it has also been documented as a complication after SARS-CoV-2 infection (47).

On MRI, it transiently involves the brainstem and basal ganglia. It is featured by regions of high T2 signal with little enhancement. Usually, these regions also show minor restriction of diffusion in DWI sequences.

### Cognitive Dysfunctions

Cognitive impairment is one of the components of a syndrome called “long-COVID” (48). Albeit there is no internationally recognized definition of long-COVID, a generally accepted time frame that differentiates the duration of the acute and post-acute infection from long-COVID is 28 days.

The causation of cognitive impairment after COVID-19 is not yet fully understood; however, studies show possible explanations. It is known that patients with SARS-CoV-2 infection have elevated risk of developing thrombovascular events.

Small vessel disease (SVD) accounts for about 80% of stroke-related dementia cases and is the most common cause of vascular cognitive impairment (49).

SVD-associated neuroimaging abnormalities of the white matter and arteriolosclerosis of cerebral microvessels can be demonstrated in about 50% of all patients with dementia.

The integrity of subcortical white matter is critically important for maintenance of cognitive function (50, 51).

Radiological demonstration after a 3-month follow-up shows that damage to white matter and disruption of functional integrity in brain regions, such as the hippocampus, was associated with memory loss in recovered COVID-19 patients (52).

Nevertheless, there are no data of specific radiological signs or findings related to cognitive disruption after SARS-CoV-2 infection that can be distinguished from SVD in older patients.

### Acute Transverse Myelitis

Acute transverse myelitis (ATM) associated with COVID-19 infection is responsible perhaps for 1.2% of all neurological complications caused by this coronavirus (53).

Roman et al., in a systematic review, presented the clinical characteristics of 43 cases of ATM in patients with COVID-19 (53). Quadriplegia (58%) secondary to lesions located within cervical/upper thoracic cord and paraplegia (42%) resulting from thoracic cord lesions were the most common clinical symptoms.

The classic symptoms in MRI are swelling and enlargement of the spinal cord and hyperintensity on T2-weighted sequences (54). Some of the lesions demonstrate enhancement after administration of a gadolinium contrast agent.

In 70–93% of patients, the lesions in the spinal cord extended into at least three spinal segments (53, 54). In isolated cases, lesions in the cervical spine extended to the brainstem or covered the entire length of the spinal cord (55, 56). Schulte et al. showed in their systematic review that transverse localization was central, frequently with extension throughout most of the transverse diameter (7/11 cases).

Clinical symptoms related to acute myelitis may appear in the 1st days from the onset of COVID-19 symptoms (32% of patients within 1–5 days) or later (68% of patients between the 2d and the 6th week) (53).

The shorter latency period may indicate a direct neurotropic effect of SARS-CoV-2 during the initial infection causing para-infectious myelitis (53). More extended latency periods may indicate a post-infectious neurological complication resulting from the host response to the virus (53).

## Manifestation of Peripheral Nervous System Complications in Radiological Findings

### Taste and Smell Dysfunctions

The most frequent specific neurological complication in COVID-19 involving peripheral nervous system are disturbances in senses of taste and smell.

According to Lechien et al. (57), the overall scope of smell dysfunctions in the studies ranged from 4.9 to 85.6%, and the most common type of smell disturbance was anosmia. Other smell abnormalities reported were hyposmia, phantosmia, and parosmia.

Similarly, the range of taste disturbances noticed was 0.3–88.8%, and the most commonly reported were dysgeusia and ageusia.

Manifestation of such complications can be captured in radiological imaging. According to data reported by Kandemirli et al. (58), changes can be shown in both tomographical examinations.

On CT, olfactory cleft opacification was seen in 73.9% of cases with a mid and posterior segment dominance.

On MRI, 91.3% of case abnormalities reveal in olfactory bulb signal intensity in the form of diffusely increased signal intensity, scattered hyperintense foci, and/or microhemorrhages.

### Guillain–Barré Syndrome

Guillain–Barré syndrome (GBS) is a heterogeneous group of autoimmune polyradiculopathies that involve sensory, motor, and autonomic nerves. It is the most common cause of rapidly progressive flaccid paralysis.

Most cases of GBS are usually preceded by upper respiratory tract infections or diarrhea 1–3 weeks before their onset, most commonly caused by *Campylobacter jejuni*. Other cases can be induced by a recent surgery, lymphoma, or active systemic lupus erythematosus.

However, recent studies show that SARS-Cov-2 infection is also a possible trigger of GBS (2).

On MRI, typical findings in Guillain–Barré syndrome are surface thickening and contrast enhancement on the conus medullaris and the nerve roots of the cauda equina.

Anterior nerve roots are considered to be the most common site of enhancement in GBS, though enhancement of the posterior nerve roots can also be observed.

The facial nerve is the most commonly affected cranial nerve.

It is essential to administer a gadolinium contrast medium when no abnormalities can be spotted in non-contrast sequences, but the diagnosis is suspected.

### Miller–Fisher Syndrome

MFS is a regional variant of Guillain–Barré syndrome and is described by cranial nerve involvement and the characteristic triad of symptoms—of ataxia, areflexia, and ophthalmoplegia.

There are studies and case reports that show that COVID-19 may cause MFS (25).

Though MRI is usually performed to diagnose patients with symptoms of MFS, there can be no abnormalities found. If radiological signs are present, usually enhancement of multiple cranial nerves is reported, just like it can be observed in Guillain–Barré syndrome.

### Others

Alongside GBS and its variants, post-infectious immune-mediated neuropathies secondary to COVID-19 have been reported (59, 60). In addition to post-infectious peripheral inflammatory neuropathies, peripheral nerve injury can occur as a consequence of hospitalization for COVID-19 (e.g., positioning-related neuropathy) or secondary to the treatment for COVID-19 (e.g., nerve entrapment secondary to hematoma) (61).

Radiological changes in the course of peripheral neuropathy are etiologically non-specific (62). Nerve enlargement and loss of bundle architecture are typical features (62). MRI shows an increase in signal intensity, with possible post-contrast enhancement (62). Ultrasound examination shows a decrease in the echogenicity of the nerve (62). As a result of the denervation, muscle swelling (subacute phase) and fatty atrophy (chronic phase) are observed (62).

## Radiological Findings in Neurological Complications

According to Radmanesh et al. (26), the most common abnormality found in radiological images were non-specific white matter changes (hypodensity on CT or T2 hyperintensity on MRI, often attributed to microangiopathy) (55.4%), chronic infarct (19.4%), acute or subacute infarcts (5.4%), and acute intracranial hemorrhage (4.5%).

Kremer et al. proposed a classification of neuroradiological findings in COVID-19 patients (5). In this categorization, radiological abnormalities are divided into three groups: (1) infarcts, (2) encephalitis, and (3) leptomeningeal enhancement.

Furthermore, the group of encephalitis was divided into (a) limbic encephalitis, (b) cytotoxic lesions of the corpus callosum (CLOCC), (c) acute disseminated encephalomyopathy (ADEM), (d) acute hemorrhagic necrotizing encephalopathy, and (e) other types of encephalitis.

## Summary

As the COVID-19 pandemic has been advancing, a growing number of complications from multiple systems are being observed. There is a general consensus that this disease is not a respiratory illness, but a systematic one.

As we presented in this article, a vast number of neurological symptoms can emerge from this viral infection and still not all complications involving nervous system are fully discovered.

As symptoms from the nervous system may precede the symptoms from the respiratory system, appear in the acute or subacute phase of the disease, or show up in the later stages of the infection or even months after recovery, it is crucial to take care of such patients holistically and precisely monitor them during the control visits.

Especially in case of the presence of nervous system implication, using radiological imaging techniques to monitor the emerging onset of various symptoms is crucial to assess the severity and scope of involvement.

The growing number of new SARS-CoV-2 infection all over the world statistically indicates that the number of neurological complications and reports of such manifestations will be growing accordingly.

Therefore, it is so important to collect feedback and identify as many cases as possible in order to allow early recognition and faster diagnosis.

Although there are not many radiological signs of neurological complications specific to COVID-19, the presence of group of symptoms may be crucial to distinguish particular syndromes and implement the proper treatment.

## Author Contributions

BM and PW contributed to conception, design of the study, and wrote the manuscript. KD and MDȩ organized the database and figures as well as performed literature search. KS contributed to the conception and design of the paper, collected the data, performed part of the analysis, the primary and final version of the manuscript, and also to the financing of the publication. JW and MDo supervised the work on the article and corrected the first draft of the manuscript. All authors contributed to the article and approved the submitted version.

## Conflict of Interest

The authors declare that the research was conducted in the absence of any commercial or financial relationships that could be construed as a potential conflict of interest.

## Publisher's Note

All claims expressed in this article are solely those of the authors and do not necessarily represent those of their affiliated organizations, or those of the publisher, the editors and the reviewers. Any product that may be evaluated in this article, or claim that may be made by its manufacturer, is not guaranteed or endorsed by the publisher.
